# Sporulation kinetics of *Bacillus subtilis* under pH shift conditions: impact on spore properties and gene expression

**DOI:** 10.1128/aem.01101-25

**Published:** 2025-09-16

**Authors:** Kaoutar Hafdane, Noémie Desriac, Clément Trunet, Yvan Le Marc, Anne-Gabrielle Mathot, Louis Coroller

**Affiliations:** 1 Laboratoire Universitaire de Biodiversité et Écologie Microbienne, Univ Brest, INRAE 27002, Quimper, France; 2 ADRIA Développement, UMT ACTIA Transispore 543631 , Quimper, France; University of Georgia Center for Food Safety, Griffin, Georgia, USA

**Keywords:** modeling, heat resistance, germination abilities, environmental conditions, sporulation genes, RT-qPCR

## Abstract

Bacterial spores are highly resistant to environmental stresses, which limits the efficiency of decontamination processes and increases the risk of spoilage and foodborne illness. While pH is a key factor influencing sporulation efficiency and spore properties, current predictive models are typically based on static conditions, limiting their relevance to food processing environments where pH can fluctuate dynamically. In this study, we investigated the effect of a single-step pH shift on *Bacillus subtilis* sporulation using an integrated modeling experimental approach. Cultures were grown in bioreactors at pH 7.0 and subjected to acidification shifts to within the growth-permissive range at pH 5.5 (pH 7.0–5.5) or below the growth limit at pH 4.0 (pH 7.0–4.0). A growth-sporulation model was applied to describe kinetics, and spores were evaluated for heat resistance and germination. Gene expression analysis was performed to monitor transcriptional responses related to sporulation, stress adaptation, heat resistance, and germination. The results showed that the shift to pH 5.5 led to delayed but successful sporulation, with prolonged expression of sporulation genes. By contrast, a shift to pH 4.0 completely blocked sporulation, with key genes becoming transcriptionally silent. While spores obtained at pH 7.0 or pH 7.0–5.5 conditions showed similar heat resistance, those from the pH 7.0–5.5 condition germinated more efficiently in L-alanine suspension. By combining gene expression analysis with sporulation kinetics, this study contributes to a better understanding of the physiological responses underlying model parameters. The approach provides a preliminary basis for future modeling of more complex environmental conditions, including multi-step pH fluctuations relevant to food processing.

**IMPORTANCE:**

Sporulation in *Bacillus subtilis* plays a critical role in food spoilage, as spores are highly resistant to environmental stresses. Environmental factors, particularly pH, significantly affect spore properties such as heat resistance and germination. While most studies focus on static pH, real-world food environments often experience fluctuating pH due to processes such as fermentation, microbial activity, and treatment processes. This gap in knowledge limits predictive microbiology models aimed at managing spore-forming bacteria in food. This study addresses this by examining how pH shifts influence *B*. *subtilis* sporulation and alter spore properties. By combining experimental data with gene expression analysis of key genes. This research provides a baseline for predicting sporulation under more realistic and dynamic environmental conditions encountered in food processing.

## INTRODUCTION

Sporulating bacteria can produce highly resistant spores, enabling them to survive extreme environmental conditions (1, 2). This characteristic makes them either beneficial or problematic, depending on the context. While it can be useful in industrial applications, such as enzyme production and probiotics, it leads to significant challenges for the food industry, such as foodborne pathogens or spoilage agents (1, 3–7). Their persistence in various environments, particularly during food processing and in industrial settings, underscores the importance of understanding and predicting the sporulation process to manage these risks effectively (1, 6). Several factors, including nutrient availability, pH, water activity, and temperature, affect sporulation kinetics, the quantity of spores produced, and their properties, such as their germination ability and resistance (8–12). Among these, pH has been shown to impact both the maximum specific growth rate and sporulation rate of *Bacillus*. Sporulation yield is higher at the optimal growth pH and decreases as the pH deviates from this optimum, leading to an extended sporulation process (13–17).

Beyond its role in sporulation kinetics, pH profoundly affects the biochemical characteristics of spores, influencing their structure, surface properties, adhesion potential, resistance, and germination ability. It affects germination efficiency, with optimal pH values generally promoting faster and more complete germination, while extreme acidic or alkaline conditions reduce germination efficiency (18). The sporulation pH also modulates spore resistance: acidic conditions often enhance heat resistance, while chemical resistance, such as to hydrogen peroxide, tends to decrease with increasing pH (15, 19–22). In addition, pH influences spore surface properties; spores formed at low pH are typically more hydrophobic and adhesive, increasing their persistence on industrial surfaces (23). These phenotypic variations are underpinned by molecular changes in spore composition, including altered levels of calcium dipicolinate (Ca-DPA), water content, and specific protective proteins, all of which contribute to the spores’ ability to withstand harsh environments (14, 16, 18, 24–29).

In food processing environments, pH is not static but fluctuates due to fermentation, acidification, microbial activity, or alkaline treatments, creating dynamic conditions (30–32). These fluctuations affect spore formation, heat resistance, and germination, ultimately impacting microbial survival in food matrices.

Predictive microbiology provides tools to model bacterial behavior under varying environmental conditions. While bacterial growth and spore resistance models under static conditions are well established (17, 19, 33, 34), models addressing sporulation under dynamic conditions remain incomplete. To address this gap, the present study builds upon the model developed by Gauvry et al. (17), adapting it to simulate sporulation kinetics under single-step pH shifts. Although these abrupt changes are not fully representative of gradual fluctuations observed in complex food matrices, they constitute a necessary first step toward capturing more realistic, multi-phase pH dynamics in future modeling work.

In addition to modeling sporulation, we aim to assess the properties of the spores produced under these conditions, specifically their heat resistance and germination abilities. Furthermore, we will investigate the molecular mechanisms of sporulation. This includes analyzing key sporulation genes, acid stress, DNA damage, and genes associated with spore properties. Understanding the influence of pH shifts on sporulation gene expression during sporulation will enhance our knowledge of its regulation and contribute to validating our model while explaining the observed differences in spore properties.

For this study, *Bacillus subtilis* was selected as a model organism due to its well-characterized genetic system, which is essential for measuring gene expression (35, 36). Beyond its role as a model bacterium, *B. subtilis* is a significant food contaminant, capable of withstanding high temperatures and acidic environments. Its persistence in food products can lead to spoilage, with high contamination rates reported in fruit juices, animal feeds, dairy products, and dairy alternatives (37–41).

## MATERIALS AND METHODS

### Bacterial strain

*Bacillus subtilis* BSB1, a trp^+^ derivative of *B. subtilis* 168 (42, 43), was kindly provided by the Micalis Institute at the French National Institute for Agricultural Research (INRAE) center in Jouy-en-Josas, France. This strain is one of the most extensively studied species within the *Bacillus* genus (35). The strain was stored at −80°C in 1 mL aliquots of Brain Heart Infusion (BHI, Biokar Diagnostics, Beauvais, France), mixed with 25% glycerol (vol/vol) at a concentration of approximately 10^7^ CFU.mL^−1^. A 250 mL flask containing 100 mL of BHI was inoculated with 1 mL of the stock suspension and incubated for 8 h at 37°C under agitation at 100 rpm. From this pre-culture, 1 mL was transferred into a 100 mL flask of BHI broth under the same culturing conditions for 12 h. A second 100-fold dilution was then performed under the same conditions in 100 mL of BHI for 6 h. Spores were assumed to be the heat-resistant cells to a heat treatment of 80°C for 10 min using a capillary method (19). The final cell concentration in the culture was approximately 10^8^ CFU.mL^−1^, while the spore concentration was below the detection threshold for enumeration (<2 log_10_ CFU.mL^−1^). Glycerol was then added to the culture at a final concentration of 25% vol/vol and stored at −80°C in 1 mL cryovials.

### Culture conditions and sample collection

Vegetative cells of *B. subtilis* BSB1 were initially inoculated from the cryovials into bioreactors (Minifors 2, Infors AG, Bottmingen, Switzerland) at an approximate concentration of 1,000 CFU.mL^−1^. Bioreactors were equipped with an integrated temperature probe and a pH electrode (EasyFerm Plus PHI ARC 325, Hamilton, Bonaduz, Switzerland). The pH probe has a resolution of 0.01 pH units, a zero-point tolerance of 0 ± 20 mV (approximately ±0.34 pH units), and was calibrated before each run using pH 4.0 and pH 7.0 buffers. The temperature probe has a precision of ±0.1°C. The bioreactors contained 3 L of BHI, supplemented with sporulation salts (CaCl_2_·H_2_O, 8.0 mg.L^−1^, and MnSO_4_·H_2_O, 1.5 mg.L^−1^) (44). Agitation was maintained at 250 rpm with an aeration rate of 1.5 L.min^−1^. The temperature was maintained at 37.0°C throughout the experiments. The pH was adjusted using 2 M sodium hydroxide (NaOH) or 2 M hydrochloric acid (HCl) following the conditions outlined in Table 1. All the parameters were maintained at the desired setpoint using an Eve bioprocess platform software (AG, Bottmingen, Switzerland). This system allowed real-time correction to ensure parameter stability throughout the growth and sporulation kinetics.

**TABLE 1:** Static and various pH shift conditions tested

Stage 1, for 16 h (t_0_–t_16 h_)	Stage 2, for 134 h (t_16 h_–t_150 h_)	Condition
pH 7.0	pH 7.0	Static
pH 7.0	pH 5.5	Acidification within the growth pH range
pH 7.0	pH 4.0	Acidification outside of the growth pH range

The static condition at pH 7.0 is referred to as “pH 7.0”*.* For the pH shift conditions, cultures were first incubated at pH 7.0 for the first 16 h, after which the pH was shifted to either 5.5 or 4.0. This time point corresponds to the end of the exponential phase, allowing full expression of growth under initial conditions and enabling the modeling of sporulation dynamics independently from ongoing growth. The pH shift conditions are defined as follows: (i) “pH 7.0–5.5” corresponds to an acidification from pH 7.0 to 5.5, occurring within the permissible pH range for *B. subtilis* growth, (ii) “pH 7.0–4.0” corresponds to an acidification from pH 7.0 to 4.0, occurring outside the permissible pH range for *B. subtilis* growth. It is important to note that the pH shift takes 24 min to stabilize at pH 5.5 and 55 min at pH 4.0. Each condition was tested in duplicate to ensure reproducibility. The culture medium was previously set to the studied conditions before inoculation. For each condition, at specific intervals during the experiment, 2 mL samples were taken for bacterial enumerations (total count and spore count), and 2 mL samples for gene expression analysis. Directly after the end of each batch, 2 L of culture was collected and centrifuged (8,000 × *g*, 4°C, 10 min). The cell pellets were washed with autoclaved distilled water and re-centrifuged (8,000 × *g*, 4°C, 10 min). Finally, the pellets were resuspended in autoclaved distilled water and stored in aliquots at a concentration of approximately 10⁸ CFU.mL⁻¹ at 4°C. These stocks were maintained for at least 2 months before heat treatment, and germination tests were performed.

### Monitoring growth and sporulation kinetics

The 2 mL samples taken periodically from the bioreactors for monitoring growth and sporulation were serially diluted in buffered peptone salt broth (PS; Clearline, Dominique Dutsher, France). Samples were then plated onto brain heart agar (BHA; Biokar Diagnostics, Beauvais, France) and incubated overnight at 37°C. Total and heat-resistant cells, assumed to be spores, were enumerated after a 10 minute heat treatment at 80°C using the capillary method. The detection limit was 2 log_10_ CFU.mL^−1^ (19).

#### Kinetic model of growth and sporulation

The growth and sporulation model consists of two modules. The first describes vegetative cell growth, while the second describes sporulation kinetics based on growth dynamics.

##### Vegetative cell growth model

Vegetative cell growth follows the modified logistic model of Kono (1968), later adapted by Rosso et al. (34, 45), as shown in equation 1:


(1) $$In\;N\left(t_{i}\right)=\left\{ \begin{array}{ll} \ln N_{0}, & t_{i}<\lambda \\ \ln\left(\frac{N_{\max}}{\left(1+\frac{N_{\max}}{N_{0}}\right)\exp\left(-\mu_{\max}\left(t_{i}-\lambda \right)\right)}\right), & t_{i}\geq \lambda \end{array}\right.$$

where *N_0_* is the concentration of the inoculum (CFU.mL^−1^), *λ* is the lag before the growth phase (h), *µ*_max_ is the maximum specific vegetative growth rate (h^−1^), and *N*_max_ is the maximum concentration of total cells (CFU.mL^−1^). Specifically, *N*_max_ corresponds to the maximum concentration of vegetative cells reached during the stationary phase. Once the first spore appears, *N*_max_ represents the total cells (both spores and the remaining vegetative cells that have not differentiated into spores).

##### Sporulation model

At each time of culture, the vegetative cells have a probability of committing to sporulation over time *P(t_i_*). It is described with the normal (or Gaussian) probability density, which was weighted by the maximum proportion *P*_max_ of the vegetative cells to sporulate (17) (equation 2):


(2) $$P\left(t_{i}\right)=P_{\max}\times \left[\frac{1}{\sigma \times \sqrt{2\pi }}\times \exp\left(-0.5\times {\left(\frac{t_{i}-t_{\max}}{\sigma \times \sqrt{2}}\right)}^{2}\right)\right]$$

where *P(t_i_)* is the probability of sporulation at time *t_i_* (h^−1^), *P*_max_ is the maximum proportion of vegetative cells forming spores (unitless), *t*_max_ is the time of maximum sporulation probability (h), and *σ* is the standard deviation around *t*_max_ (h).

Once a vegetative cell commits to sporulation, the differentiation process requires some time to complete sporulation (*t_f_*). We assumed a constant time for sporulation (*t_f_*) of 7 h, based on prior research by Piggot and Hilbert (2004) (46). Their study demonstrated that *Bacillus subtilis* spore formation takes about 7 h at 37°C, beginning with the activation of the master transcription regulator for entry into sporulation, Spo0A, and ending with the release of the spore by the mother cell. We defined a mature spore as a cell that is resistant to a heat treatment of 10 min at 80°C. Based on these assumptions, the sporulation kinetics can be defined by equation 3 (17).


(3) $$S\left(t_{i}\right)=\left\{ \begin{array}{ll} 0, & t_{i}<t_{f}\\ S\left(t_{i-1}\right)+\left(\left[N\left(t_{i}-t_{f}\right)-S\left(t_{i-1}\right)\right]\times P\left(t_{i}-t_{f}\right)\right), & t_{i}>t_{f} \end{array}\right.$$

where *N(t_i_ − t_f_*) are the total cells at time *t_i_ − t_f_* (CFU.mL^−1^), *S(t_i_*_− 1_*)* are the spores at time *t_i_*_− 1_ (CFU.mL^−1^), and *P(t_i_ − t_f_*) is the probability of vegetative cells committing to sporulation at time *t_i_ − t_f_* (h^−1^).

### Estimation of growth and sporulation parameters under static conditions

Growth and sporulation parameters were estimated by fitting the experimental data to the growth-sporulation model described above. These parameters included the maximum specific growth rate (*µ*_max_), lag phase duration (*λ*), maximum sporulation probability (*P*_max_), time at maximum sporulation probability (*t*_max_), and standard deviation around *t*_max_ (*σ*). The fitting procedure used nonlinear regression to minimize the sum of squared errors (*SSE*) defined by equation 4:


(4) $$SSE=\sum (y_{i}-{\hat{y}}_{i}{)}^{2}$$

where *y_i_* is the experimental data for the concentration of total cells or spores (log_10_ CFU.mL⁻¹) and *ŷ_i_* is the corresponding value estimated by the model (log_10_ CFU.mL⁻¹).

Optimization was performed using the fmincon function (MATLAB, Optimization Toolbox Release 2021b, The MathWorks, Inc., Natick, MA, USA). The 95% confidence intervals were calculated from the linear regression of the parameter estimates (nlparci, MATLAB, Statistics Toolbox Release 2021b, The MathWorks, Inc.). For parameter estimation, multiple model configurations were tested: (i) a configuration where *N_0_* and *N*_max_ were shared between replicates, (ii) a configuration where both replicates were adjusted together with separate initial (*N_0_*) and maximum (*N_max_*) cell concentrations, and (iii) a configuration with separate adjustments for each replicate individually.

For each experimental growth-sporulation kinetic data set (total cell and spore concentrations) under static conditions (37.0°C and pH 7.0), both the observed and fitted kinetics were plotted. The fitting quality of each model configuration was evaluated using the root mean square error (RMSE) and the corrected Akaike information criterion (AICc), with lower values indicating a better model fit (equations 5 and 6). To ensure a comprehensive assessment, model performance was evaluated in three ways: (i) separately for growth kinetics (RMSE*_N_* and AICc*_N_*), (ii) separately for sporulation kinetics (RMSE*_S_* and AICc*_S_*), and (iii) globally for both kinetics combined (RMSE_tot_ and AICc_tot_).

The RMSE is calculated as follows:


(5) $$RMSE=\sqrt{\frac{1}{n}\sum_{i=1}^{n}{\left(y_{i}-{\hat{y}}_{i}\right)}^{2}}$$

where *y_i_* is the experimental data for the concentration of total cells or / and spores (log_10_ CFU.mL⁻¹) and *ŷ_i_* is the corresponding value estimated by the model (log_10_ CFU.mL⁻¹).

The AICc is calculated as follows:


(6) $$AICc=2k-2In(L)+\frac{2k(k+1)}{n-k-1}$$

where *k* is the number of estimated parameters, *L* is the maximum likelihood of the model, and *n* is the number of experimental observations.

### Describing the impact of environmental parameters on growth and sporulation

The effects of temperature on bacterial growth and sporulation were described with the cardinal model (CM-function) of Rosso et al*.* (34), with equation 7 and pH with an Aryani-type model extended to supra-optimal pH (47), with equation 8, and the interaction between the pH and temperature factors with equation 9:


(7) $$CM_{2}(T)=\left\{ \begin{array}{ll} \frac{\left(T-T_{\max}\right)\times {\left(T-T_{\min}\right)}^{2}}{\left(T_{opt}-T_{\min}\right)\times \left[\left(T_{opt}-T_{\min}\right)\times \left(T-T_{opt}\right)-\left(T_{opt}-T_{\max}\right)\times \left(T_{opt}+T_{\min}-2T\right)\right]}, & T_{\min}<T<T_{\max}\\ 0, & T\leq T_{\min}\text{ or }T\geq T_{\max} \end{array}\right.\;$$

(8) $$CM(pH)=\left\{ \begin{array}{ll} \left(1-10^{2\left(pH_{\min}-pH\right)}\right)\cdot \left(1-10^{2\left(pH-pH_{\max}\right)}\right), & pH_{\min}<pH<pH_{\max}\\ 0, & pH\leq pH_{\min}\text{ or }pH\geq pH_{\max} \end{array}\right.\;$$

(9) $$Y_{\max}(T,pH)=Y_{\text{opt }}\times CM_{2}(T)\times CM(pH)$$

where *T* represents the temperature (°C). *T*_max_, *T*_min_, and *T*_opt_ correspond to the maximum, minimum, and optimal temperature values at which the strain was able to grow. Similarly, pH_max_ and pH_min_ represent the maximum and minimum pH values for growth. The temperature cardinal values used as input were obtained from a previous study of Gauvry et al., while pH_max_ and pH_min_ were estimated using the Aryani model and are shown in Table 2 (17, 47). Y_max_ is the growth and sporulation parameters (*μ*_max_, 1/*λ*, −log₁₀(*P*_max_), *t*_max_, and *σ*).

**TABLE 2:** Cardinal values of *B. subtilis* BSB1: minimum temperature (*T*_min_), optimal temperature (*T*_opt_), and maximum temperature (*T*_max_) for growth, as well as minimum pH (pH_min_), and maximum pH (pH_max_) for growth (17)

Cardinal values	*Bacillus subtilis BSB1*
*T*_min_ (°C)	5.5
*T*_max_ (°C)	55.7
*T*_opt_ (°C)	46.9
pH_min_	4.58
pH_max_	9.10

### Simulation of growth and sporulation kinetics under pH shift conditions

The effect of the static condition (37.0°C and pH 7.0) was calculated using the cardinal model (CM-function), detailed above. This effect was then used to determine the corresponding parameters under optimal conditions (46.9°C, pH 6.9), to estimate the corresponding parameters at the two pH levels used in the pH shift conditions. To model the evolution of total cells and spores under pH shift conditions, we assumed that the models developed under constant conditions remain valid under conditions with two pH shifts. To ensure complete vegetative growth before the environmental transition, the pH shift was introduced at the end of the exponential phase, that is, once the maximum vegetative cell concentration had been reached. This design allowed full expression of the growth capacity under initial conditions and enabled the modeling of sporulation dynamics independently from ongoing growth.

For two successive environmental conditions, A and B, the probability of initiating sporulation first follows the parameters *P*_max_ (*A*), *t*_max_ (*A*), and *σ* (*A*). At the time of the environmental shift (*t*_shift_), we assumed that the cells could be classified into three states: (i) spores already formed, (ii) vegetative cells committed to sporulation, which will yield spores after the sporulation formation time (*t_f_*) with the parameters of the first condition, and (iii) non-committed vegetative cells, which will enter sporulation following the probability imposed by the new condition (B). The probability of sporulation commitment in condition B originates from the cumulative percentage of committed cells at the time of transition. The initial concentrations of vegetative cells (*N*_0_ and *N*_max_) used in the simulations were the experimentally observed values.

For each experimental growth-sporulation kinetic data set (total cell and spore concentrations), both the observed and simulated kinetics were plotted. The goodness of the simulation by the model was evaluated separately for vegetative cells and spores using the RMSE and the mean relative error (RE_mean_) (equation 10), with lower values indicating a good simulation. To ensure a comprehensive assessment, model performance was evaluated in three ways: (i) separately for growth kinetics (RMSE*_N_* and RE*_N_*), (ii) separately for sporulation kinetics (RMSE*_S_* and RE*_S_*), and (iii) globally for both kinetics combined (RMSE_tot_ and RE_tot_).

The RE_mean_ is calculated as follows:


(10) $$RE_{\text{mean }}=\frac{1}{n}\sum_{i=1}^{n}\frac{y_{i}-{\hat{y}}_{i}}{y_{i}}$$

where *y_i_* is the experimental data for the concentration of total cells or spores (log_10_ CFU.mL⁻¹) and *ŷ_i_* is the corresponding value estimated by the model (log_10_ CFU.mL⁻¹).

### Monitoring of gene expression

The 2 mL samples taken periodically from the bioreactors for the gene expression analysis were divided into two 1 mL aliquots, which were centrifuged separately (8,000 × *g*, 4°C, 10 min). The pellets from each aliquot were resuspended in 1 mL of RNA Protect reagent (Qiagen, Courtaboeuf, France) and then incubated for 5 min at room temperature. After further centrifugation (8,000 × *g*, 4°C, 10 min), the cell pellets were frozen and stored at −80°C until RNA extractions were performed.

#### RNA extraction and cDNA synthesis

The RNA extraction and cDNA synthesis were assessed using an adapted version of the protocol detailed in the study by Desriac et al*.* (48). Briefly, thawed cell pellets were suspended in the TE buffer and disrupted by lysozyme with an activity of 8 U.mg^−1^ and proteinase K with an activity of 14,678 U.mg^−1^ for 30 min at 25.0°C. RNA was then purified using the RNeasy Mini Kit (Qiagen, Courtaboeuf, France) following the manufacturer’s instructions. To eliminate any residual genomic DNA, RNA samples were treated twice with the DNA-free Kit (Invitrogen, Thermo Fisher Scientific, Vilnius, Lithuania). The absence of genomic DNA contamination was confirmed by performing a control qPCR on RNA samples without reverse transcription. Only RNA samples showing no amplification in these controls were used for cDNA synthesis. RNA quantity and quality were assessed using a Nanophotometer N60 (Implen GMBH, Germany). An adjusted volume of total RNA corresponding to 150 ng was reverse-transcribed into cDNA in a final volume of 20 µL using an iScript cDNA synthesis kit (Bio-Rad, Mitry Mory, France) following the manufacturer’s instructions. The potential interference of spores in RNA extraction was assessed by preparing suspensions with varying spore-to-vegetative cell ratios. DNA was extracted from these samples using a DNeasy Mini Kit (Qiagen, Courtaboeuf, France) and quantified using a Nanophotometer N60. The results showed no significant differences in DNA quantity between samples with and without spores, even at spore concentrations as high as 10^8^ CFU.mL^−1^.

#### Primer design and quantitative PCR

cDNA amounts were quantified by qPCR in a final volume of 25 µL containing iQ SYBR Green Supermix (Bio-Rad), 250 nM of each primer, and 2.5 µL of cDNA. A total of 18 target genes were analyzed, including six key sporulation genes, four acid stress and DNA damage-related genes, four genes involved in the regulation of spore properties, and four reference genes (Table 3). The selection of these genes was based on literature research (36, 46, 49). To ensure a meaningful comparison of gene expression across conditions, sample selection was carefully designed. Time points at *t =* 0 h and *t =* 14 h of each experiment were included as common reference points before the pH shift, allowing us to verify whether gene expression followed a similar initial pattern across all conditions. In addition, several other time points were selected during the sporulation kinetics, corresponding to equivalent spore concentrations across all tested conditions. This approach ensured that differences in gene expression were not merely due to variations in sporulation progress but rather reflected pH-dependent regulatory effects. Primer sequences were designed using the PrimerQuest Tool (IDT), and their specificity was verified using the SnapGene tool by comparing them against the complete *Bacillus subtilis* genome. The stability of primer secondary structures, including hairpin, dimer, and cross-dimer formations, was evaluated by calculating the Gibbs free energy (ΔG) using the Beacon Designer free edition tool. Primers with stable secondary structures, indicated by the ΔG value recommended in the software’s technical note, were avoided. To prevent interference from secondary structures during annealing, both the cDNA and PCR product folding structures were analyzed in the mFold Web Server (default settings). Experimental validation was assessed using a melt curve analysis and a dilution series of genomic DNA, respectively. Only primers with an efficacy of 90%–100% with R^2^ ≥0.980 were used in this study. To minimize inter-run variations, three calibrator samples consisting of three decimal dilutions of a known quantity of genomic DNA were used to adjust all quantitative cycle Cq values (Bio-Rad CFX manager software). All the PCR experiments were conducted in technical duplicates of the two biological replicates and included blank samples containing water instead of the cDNA as no-template controls (NTC) to verify the absence of contamination or primer-dimer formation.

**TABLE 3:** Target genes selected based on literature and RT-qPCR assay details, including locus tag, product, and biomarker type (36, 46, 49)

Gene	Locus tag	Product	Biomarker type	Efficiency (%)	R²	Intercept	Amplicon size (bp)	Forward primer sequence	Reverse primer sequence	Cq range
*rapA*	BSU_12430	Response regulator aspartate phosphatase	Inactivation of Spo0A in low cell density	92.6	0.999	39.965	140	CGATTCCGTCCTCTTATGT	CAGCAAATCTTGGTCTTCTT	13.66–27.50
*spo0A*	BSU_24220	Phosphorelay response regulator	Sporulation initiation	90.6	0.997	39.178	143	CAGGAAGACATGGAAGTGA	TCCCTCAGCCTCTCTAAA	12.60–26.79
*spoIIE*	BSU_00640	Protein serine phosphatase, septum-associated PP2C	Asymmetric cell division	90.4	0.997	38.919	166	TCCACGCTTGACCTATCT	CACTCACAACCTCCACATC	9.14–26.84
*spoIIGA*	BSU_15310	Pro-SigE protease	Maturation of SigE (early mother cell transcription)	91.6	0.997	38.635	150	GGTGGCTTTCCAGCATTA	CTGGTTACCGGAATCAATCA	12.35–26.46
*spoIIB*	BSU_28060	Facilitator of septal dissolution	Regulation of septal peptidoglycan dissolution during engulfment	90.9	0.999	39.859	147	GTGTTTAAAGAGGACCCTAAAG	CCCTAATCCAGTGCCAAT	13.58–28.20
*spoIID*	BSU_36750	Lytic transglycosylase	Dissolution of the septal cell wall during engulfment	91.2	0.996	38.665	148	TCCCACACTCTTGGTTATAC	GGTTCGATAGACGGGAATAG	12.19–22.89
*sigK*	BSU_25760	RNA polymerase sporulation sigma factor	Late mother cell transcription	90.0	0.998	38.679	148	TCGGCTTTGTTGTTAAAGAG	TGCTCAATCAGCATGTTTC	12.09–26.07
*disA*	BSU_00880	DNA integrity scanning protein	Control of sporulation initiation under DNA damage	90.1	0.998	38.152	148	GGTTACCCTGCTTCTACTAATC	CTGCACTCGCCTCAATAA	11.28–25.47
*rsiO*	BSU_33250	Anti-SigO-RsoA	Acid stress response	94.2	0.998	38.525	149	GGCTGATGATACATAGCTTTAC	TTCTTCTGTGGTGGACAG	12.63–26.38
*des*	BSU_19180	Phospholipid desaturase	Membrane fluidity adaptation at low temperatures	91.7	0.995	39.707	143	GACAGGTGTCCTGACTTTAT	GTCTGGATGCAGCTTTATATTC	13.34–27.14
*ftsZ*	BSU_15290	cell-division initiation protein	Asymmetric cell division	94.2	0.999	38.463	148	GAAGAAAGCAAAGAGCAGAT	CAACGCCGACTGTTAATG	12.49–26.32
*gyrA*	BSU_00070	DNA gyrase (subunit A)	Reference gene	90.5	0.997	38.695	162	GAAGAATCACACGTCCTTATC	CTCTTCACCTTTAGTAGCTTTC	12.06–26.49
*tufA*	BSU_01130	Elongation factor Tu	Reference gene	90.2	0.995	38.695	145	CGTGAGCACATCCTTCTTTCTA	CAGGGAAGTCGTATTCGCTAAG	11.95–26.79
*dnaA*	BSU_00010	Replication initiation protein	Reference gene	94.0	0.997	37.732	144	TCCGACACTTGAAGACAGATTG	AGCATAACCTCGTTCGGAATATC	11.75–26.87
*spoVS*	BSU_16980	Unknown	Spore coat assembly, spore core dehydration	91.7	0.995	38.789	155	ATCGAGTCCAAATTCAGTGG	TCAAATCAACGCCGCTT	12.34–23.29
*spoVAEB*	BSU_23402	Spore germinant protein	Calcium dipicolinate uptake	92.6	0.979	41.075	149	CGGTGCAATATTAGATGG	TGCCGATTCCGATAAA	15.54–20.68
*gerAA*	BSU_33050	Ion channel protein	Germination response to alanine	92.0	0.963	39.472	155	CCGTTCCCTCCGATATTTG	CTGACGAGATTCGCTTCTAC	12.60–26.37
*gerPC*	BSU_10700	Spore germinant protein	Nutrient germinants	92.3	0.979	41.075	160	GACAGCAGGATTCACTAT	GCTCATCTCCTTTCATTTC	13.34–27.13

#### Gene expression quantification and statistical data analysis

The gene expression analysis was conducted using CFX Maestro software 1.0 (Bio-Rad Laboratories, Inc.), which automatically performs threshold setting and baseline correction. The software determined Cq values using the single threshold mode. The Cq for each sample was converted into the relative quantity of gene expression (Δ*Cq*) while accounting for primer-specific amplification efficiency, using the following formula (equation 11):


(11) $$\Delta Cq_{\left(GOI,\text{ sample }\right)}=E_{GOI}^{\left[\left(C_{q}(\text{ control })-C_{q}(\text{ sample })\right)\right]}$$

where Δ*Cq_(_*_GOI, sample_*_)_* is the relative quantity of gene expression of the sample being analyzed for a specific gene of interest GOI, *Cq*(control) is the mean *Cq* value for the sample at time = 0 h (*t_0_*) for the gene of interest and *Cq*(sample) is the mean *Cq* value for the sample, and *E*_GOI_ is the efficiency of the primer of corresponding GOI, calculated using the following formula (equation 12):


(12) $$E=\left[\left(\frac{Efficiency\;(\%)}{100}\times 0.01\right)+1\right]$$

with 100% efficiency corresponding to *E* = 2.

Δ*Cq*_(GOI, sample)_ is then converted to relative normalized expression (ΔΔ*Cq*) using the following formula (equation 13):


(13) $$\Delta \Delta Cq_{\text{(sample, GOI) }}=\frac{RQ_{\text{sample (GOI) }}}{{\left(RQ_{\text{sample (Ref 1) }}\times RQ_{\text{sample (Ref 2) }}\times \cdots \times RQ_{\text{sample (Ref n) }}\right)}^{\frac{1}{n}}}$$

where ΔΔ*Cq*_(sample,GOI)_ is the relative normalized expression of the gene of interest (GOI) in the analyzed sample, normalized to reference genes within the biological system, and RQ is the relative quantity of the studied sample. Ref refers to the selected reference genes.

The final gene expression values were calculated as log_2_(2^(*−*ΔΔ*Cq*)^) to determine fold change in expression levels.

The gene stability of all target and reference genes was evaluated using Bio-Rad CFX Manager software, which calculates the average pair-wise variation of reference genes (50, 51). Based on this analysis, *tufA*, *gyrA*, and *tfsZ* were selected as stable reference genes for normalization in subsequent qPCR analysis.

To ensure accurate quantification, minimum and maximum quantification limits of the primers were set for each gene in the Bio-Rad CFX software (Table 3). No samples had Cq values above the upper limit of quantification. However, for samples where gene expression fell below the lower quantification limit, the corresponding log_2_(2^(−ΔΔ*Cq*)^) value was replaced by the minimum assigned value specific to each gene, thereby ensuring that weak gene expression was appropriately reflected.

Following data processing, Principal Component Analysis (PCA) was performed to explore the overall structure of gene expression profiles. Separate PCAs were conducted for (i) sporulation-related genes and (ii) genes associated with spore properties (heat resistance and germination). PCA was performed using the FactoMineR and factoextra packages in R (version 4.3.0), with variables scaled to unit variance. For each PCA, qualitative supplementary variables were included to classify samples according to the condition (pH 7.0, pH 7.0–5.5, pH 7.0–4.0). In the PCA plots of individuals, time points were used as labels to visualize the temporal progression of gene expression profiles. Confidence ellipses at the 95% level were added for each pH condition. Samples collected before the pH shift (*t* ≤ 16 h) were treated separately under the category “Before shift.” Custom graphical themes were applied to improve visualization.

To complement the global PCA approach, individual gene expression kinetics were analyzed. For each sporulation gene (*rapA*, *spoIIE*, *spoIIGA*, *spoIIB*, *spoIID*, and *sigK*), the time course of expression was plotted. Mean expression levels and standard deviations were calculated separately for each condition (pH 7.0 and pH 7.0–5.5) and each time point. Bar plots with grouped bars were generated using MATLAB (R2023a) with custom scripts. Each bar represents the mean ± standard deviation across biological replicates. For each gene and time point, statistical differences in expression between the pH 7.0 and pH 7.0–5.5 conditions were assessed using unpaired two-sample t-tests. Significant differences (*P* < 0.05) were annotated with asterisks above the bars. For time points or conditions where gene expression was below quantification limits, the minimum assigned value for the gene was used in the calculation, following the same normalization strategy as for PCA.

### Heat treatment

The spores collected at the end of each batch were diluted in PS to a final concentration of around 10^7^ spores.mL^−1^. Capillary tubes of 200 µL were filled with 100 µL of spore suspension, sealed, and then immersed in a water-glycerol bath maintained at 90°C, 95°C, and 100°C. Capillary tubes were removed from the bath at appropriate time intervals and immediately cooled in a water bath for 30 s. The capillary tubes were broken, and the heat-treated spore suspensions were diluted in peptone salt broth. Volumes of 1 mL of the appropriate decimal dilutions of heat-treated spores were spread on BHA using the pour plate method. Enumeration of colonies was performed after overnight incubation at 37.0°C. All the heat inactivation kinetics were conducted in technical duplicates of the two biological replicates.

#### Modeling heat inactivation curves and statistical analysis

Heat inactivation curves were fitted with the model presented in the following equation 14 (52):


(14) $$\log_{10}N=\log_{10}N_{0}-{\left(\frac{t}{\delta }\right)}^{p}$$

where *N* is the surviving population (CFU.mL^−1^), *N*_0_ is the initial spore population (CFU.mL^−1^), δ is the time to the first decimal reduction (min), and *p* is a shape parameter (unitless).

The model was fitted to the observations (log_10_(*N*)) by minimizing the SSE using the nlinfit function in MATLAB (Optimization Toolbox Release 2021b, The MathWorks, Inc.). To ensure consistency and simplify interpretation, technical replicates were fitted simultaneously, while biological replicates were fitted separately to account for batch-induced variability. The 95% confidence intervals for the estimated parameters were computed using the nlparci function in MATLAB (Statistics Toolbox Release 2021b, The MathWorks, Inc.). The goodness of fit of the model was checked by the AICc (equation 6) and the RMSE. Lower AICc and RMSE indicate a better model fit.

To assess whether a simplified model with a fixed shape parameter *p* = 1 could be used, we compared the performance of models with and without a fixed shape parameter using the likelihood test *S_L_*, defined as:


(15) $$S_{L}=n\times \ln\left(\frac{{RSS}_{C}}{{RSS}_{U}}\right)$$

where n is the number of observations, RSS*_C_* is the residual sum of squares of the constrained model (where *p* is fixed at 1 across all inactivation curves), and RSS*_U_* is the residual sum of squares of the unconstrained model (where *p* is estimated individually for each inactivation curve).

The heat inactivation curves with and without a fixed *p*-value were considered significantly different when the likelihood ratio (*S_L_*) exceeded Pu − Pc, where Pu is the number of parameters in the unconstrained model (where *p* is not fixed), and Pc is the number of parameters in the constrained model (where *p* is fixed at 1). The critical value was determined based on the Chi-squared distribution table for Pu − Pc degrees of freedom at a significance level of *α* = 5%.

To statistically compare *D*-values (or *δ*-values) of spores produced under different pH conditions, a three-way ANOVA (factors: replicate, pH, and temperature) was conducted using the anovan function in MATLAB (Statistics Toolbox Release 2021b, The MathWorks, Inc.), followed by multiple comparisons (Tukey HSD) using multicompare (Statistics Toolbox Release 2021b, The MathWorks, Inc.). A significance level of *α* = 5% was applied.

The effect of the heat treatment temperature was quantified by the Bigelow model (equation 16) (53):


(16) $$\log_{10}D=\log_{10}D^{*}-\left(\frac{T-T^{*}}{Z^{T}}\right)$$

where *D* is the decimal reduction time at temperature *T* (min), D* is the reference *D*-value at the reference temperature (100°C) (min), *Z_T_* is the temperature increase needed to reduce *D* by one log_10_ unit (°C), and *T* is the treatment temperature (°C).

Fitting was performed using nonlinear regression with the nlinfit function in MATLAB (Statistics Toolbox Release 2021b, The MathWorks, Inc.).

### Germination kinetics monitoring by flow cytometry

#### Sub-population gating

The germination process was monitored using the protocol developed by Trunet et al*.* (54). Flasks of 100  mL of BHI or a suspension of 100 mM of L-alanine in Tris-HCl 10 mM were inoculated at a final concentration of 10^6^ spores mL^−1^. The flasks were adjusted to pH 7.14 and inoculated with spores collected from the different batches and incubated at 37°C. At specific time points during incubation, 900 µL of these suspensions was sampled and stained with 1 µL of Syto 9 dye (Molecular Probes, Life Technologies, Saint-Aubin, France). Changes over time in counts of dormant spores, germinated spores, and/or vegetative cells were quantified using a flow cytometer (Cyflow Space; Sysmex Europe Gmbh, Norderstedt, Germany) equipped with an excitement laser light source at 488  nm and three detectors: forward scatter (FSC), side scatter (SSC) detecting light emission at 488  nm, and a fluorescence light detector with a 536/40 nm filter (FL1). The software used to collect and analyze the flow cytometry data was Flomax 2.3. Spores and/or cells were analyzed at 1 µL.s^−1^ with thefollowing voltages: 109  mV (FSC), 310  mV (SSC), and 251  mV (FL1). Cell populations were analyzed until the first cell divisions, revealed by a significant increase in the total cell number during germination in BHI and after 24 h in the L-alanine suspension. All the germination kinetics were conducted in technical duplicates of the two biological replicates. Concurrently, a volume of 10 µL from each suspension was examined using phase-contrast microscopy and epi-fluorescence microscopy, employing excitation wavelengths of 470–490 nm (Microscope Olympus BX50, Olympus Optical Co., Ltd, Hamburg, Germany) to control the flow cytometry observation. Dormant spores lose their refractility as they germinate, transitioning from phase-bright to phase-dark and becoming Syto9 positive as spore DNA becomes accessible.

#### Modeling of germination and statistical analysis

The germination kinetics of spores were modeled using a modified Weibull cumulative distribution function, defined by the following equation 17:


(17) $$P_{\text{germ }}(t)=\tau_{G}\times \left(1-10^{-{\left(\frac{t}{t_{\text{germ }}}\right)}^{S_{\text{germ }}}}\right)$$

where *P*_germ_ *(t)* is the proportion of germinated spores over time, *t*_germ_ is the time taken to reach 90% of maximum number of germinated spores (min), *S*_germ_ is a shape parameter representing the scattering of germination times within the population (a higher *S*_germ_ value indicates lower scattering), and *τ_G_* is the final germination percentage, representing the maximum proportion of spores that successfully germinate.

The model was fitted to the observations by minimizing the *SSE* using the fmincon function in MATLAB (Optimization Toolbox Release 2021b, The MathWorks, Inc.). To ensure consistency and simplify interpretation, technical replicates were fitted simultaneously, while biological replicates were fitted separately to account for batch-induced variability. The 95% confidence intervals for the estimated parameters were computed using the nlparci function in MATLAB (Statistics Toolbox Release 2021b, The MathWorks, Inc.). The goodness of fit of the model was checked using the RMSE and AICc (equations 5 and 6), where lower RMSE and AICc values indicate a better model fit.

To determine whether the germination parameters (*S*_germ_, *t*_germ_, and *τ_G_*) differed significantly between experimental conditions, a multifactorial analysis of variance (ANOVA) was performed. The analysis included four categorical factors: the biological replicate (Rep 1 vs. Rep 2), the pH level during sporulation, the germination medium (BHI vs. L-alanine), and a fourth combined factor representing the interaction between sporulation pH and medium was also included to assess cross-effects. The ANOVA was conducted using the anovan function in MATLAB (Statistics Toolbox Release 2021b, The MathWorks, Inc.), followed by multiple comparisons (Tukey HSD) using multicompare (Statistics Toolbox Release 2021b, The MathWorks, Inc.). A significance level of *α* = 5% was applied.

## RESULTS

### Growth and sporulation kinetics under different pH conditions

#### Kinetics of growth and sporulation at static pH 7.0 and 37.0°C

**Fig 1:**
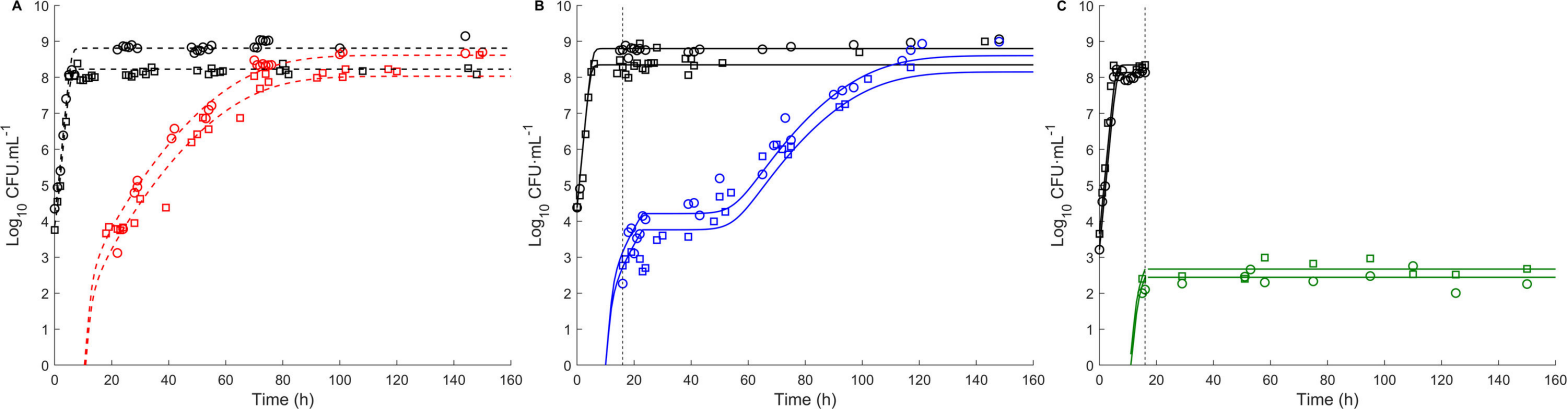
*B. subtilis* growth and sporulation kinetics under static and pH shift conditions. (**A**) At a pH 7.0 condition, growth and sporulation kinetics were fitted to experimental data under static conditions (37.0°C, pH 7.0), as represented by the dashed lines. (**B, C**) Growth and sporulation were simulated under shift conditions based on parameters estimated at pH 7.0 (solid lines). For the two pH shift conditions, the pH was initially set at 7.0 for the first 16 h. In (**B**) at the pH 7.0–5.5 condition, the pH was shifted to 5.5. In (**C**) at the pH 7.0–4.0 condition, the pH was shifted to 4.0. Experimental data points represent the mean values of technical duplicates for two biological replicates (O, □), with total cells in black and thermoresistant spores in red (**A**), blue (**B**), and green (**C**). Dashed vertical lines indicate the time point at which the pH shift occurred.

The results showed that the configuration, joint adjustment of both replicates adjusted together with independent *N*_0_ and *N*_max_ values, provided the best compromise between fit quality and parameter parsimony (Fig. 1A). It provided the lowest AICc value of 40.86 for total cells and spores, as shown in Table 4, with the most accurate estimates for growth and sporulation parameters (Table 5).

**TABLE 4:** Statistical comparison of different model configurations for growth and sporulation of *B. subtilis* at static pH 7.0 and 37.0°C^*a*^

	Growth		Sporulation		Growth and sporulation	
	RMSE_*N*_	AICc_*N*_	RMSE_*S*_	AICc_*S*_	RMSE_tot_	AICc_tot_
Separate fit—Replicate 1	0.20	−5.85	0.25	5.50	0.22	0.45
Separate fit—Replicate 2	0.16	−21.34	0.41	29.88	0.29	31.51
Joint fit—shared parameters	0.38	61.25	0.43	54.30	0.40	116.07
Joint fit—shared parameters, except *N_0_* & *N*_max_	0.19	−23.56	0.37	40.75	0.28	40.86

^*a*^ This table presents the RMSE and the AICc, calculated separately for growth kinetics* (*RMSE*_N_* and AICc*_N_), *sporulation kinetics RMSE*_S_* and AICc*_S_*, and the combined growth and sporulation kinetics (RMSE*_N_* and AICc_tot_*).*

**TABLE 5:** Estimated values and standard deviations of growth and sporulation parameters at static pH of 7.0 and 37.0°C, for all shared parameters except *N_0_* & *N*_max_

Parameter	Value ± standard deviation
*N_0_* (ln CFU.mL⁻¹)—Replicate 1	9.74 ± 0.53
*N_0_* (ln CFU.mL⁻¹)—Replicate 2	8.91 ± 0.51
Lag phase (*λ*) (h)	0.33 ± 0.40
*µ*_max_ (h^−1^)	1.90 ± 0.12
*N*_max_ (ln CFU.mL⁻¹)—Replicate 1	20.28 ± 0.11
*N*_max_ (ln CFU.mL⁻¹)—Replicate 2	18.94 ± 0.11
*P*_max_	1.00 ± 0.21
*t*_max_ (h)	74.65 ± 3.07
*σ* (h)	14.39 ± 0.71

#### Kinetics of growth and sporulation under pH shift conditions

For the pH 7.0–5.5 condition, sporulation initially followed the same trend as under static conditions from 0 to 16 h. At this point, corresponding to the end of the exponential growth phase, a pH shift from 7.0 to 5.5 was applied. Following the shift, spore formation yield slowed down to 8.5 × 10^3^ CFU.mL^−1^ for ~22 h before increasing again, reaching 4.2 10^8^ CFU.mL^−1^ after 125 h (Fig. 1B). This predicted 22 hour lag time was due to the impact of the pH decrease from 7.0 to 5.5. The estimated *t*_max_ at pH 5.5 is delayed by 15 h compared to pH 7.0 (Table 6), and the spore formation time is 7 h, corresponding to the observed 22 hour delay after the shift in the pH 7.0–5.5 condition. The low RMSE and RE values confirm the model accuracy in simulating growth and sporulation (Table 7).

**TABLE 6:** Estimated and predicted sporulation parameters under different conditions

	Estimation at 37.0°C, pH 7.0	Prediction at 37.0°C, pH 5.5	Prediction at 37.0°C, pH 4.0
*P*_max_	1.00 ± 0.21	1.00	0
*t*_max_ (h)	74.65 ± 3.07	89.59	0
*σ* (h)	14.39 ± 0.71	14.19	0

**TABLE 7:** Statistical evaluation of the predicted model for growth and sporulation of *B. subtilis* under pH shift conditions, pH 7.0–5.5 and pH 7.0–4.0*^a^*

Condition	pH 7.0–5.5	pH 7.0–4.0
RMSE*_N_*	0.2607	0.3387
RMSE*_S_*	0.4886	0.2387
RMSE_tot_	0.3878	0.3023
RE*_N_*	−0.0058	0.0210
RE*_S_*	−0.0132	−0.0305
RE_tot_	−0.0094	−0.0009

^*a*^ This table presents the RMSE, and the RE for growth kinetics (RMSE*_N_*, RE*_N_*), sporulation kinetics (RMSE*_S_*, RE*_S_*), and growth and sporulation kinetics together (RMSE_tot_, RE_tot_).

For the pH 7.0–4.0 condition, the total cell concentrations initially increased, while spore concentrations reached approximately 2 log_10_ CFU.mL^−1^ after 16 h. After the pH shift, a significant cellular inactivation of vegetative cells was observed, resulting in total cell counts exceeding spore counts by less than 0.5 log_10_ CFU.mL^−1^ (see Fig. 1C). Since the growth model assumes a constant viable population and does not include cell inactivation, the decline in vegetative cell concentration following the shift could not be simulated and was therefore not modeled. The sporulation was completely inhibited under these conditions, with the spore concentration remaining unchanged after the pH shift to 4.0 (Fig. 1C). This observation was consistent with the model prediction of a null sporulation probability (*P*_max_ = 0) at pH 4.0. The low RMSE and RE_mean_ values confirm the model accuracy in simulating growth and sporulation (Table 7).

### Effect of sporulation under pH shift conditions on the expression of genes related to sporulation, acidic stress, and DNA damage

**Fig 2:**
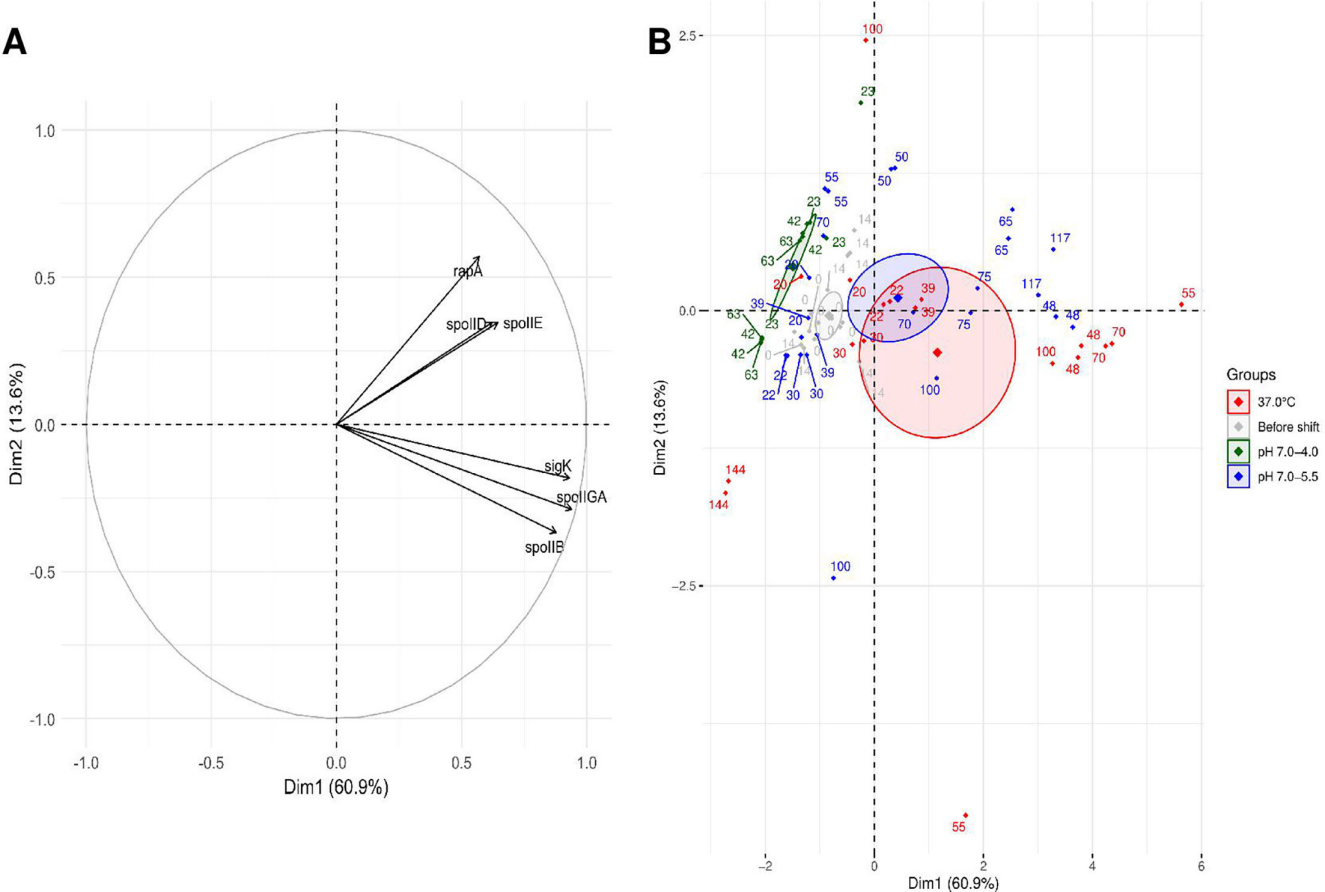
PCA of gene expression during sporulation under different pH conditions. (**A**) Correlation circle showing the contributions of selected sporulation genes to the first two principal components (Dim1 and Dim2). (**B**) PCA score plot showing the distribution of samples before (gray) and after the pH shift under different pH conditions: pH 7.0 (red), pH 7.0–5.5 (blue), and pH 7.0–4.0 (green). Each point corresponds to one of the two biological replicates at a given time point and represents the mean of two technical replicates. Time points are labeled (0–144 h, depending on the time at which the sampling was performed), and 95% confidence ellipses illustrate group dispersion by sporulation condition.

The PCA showed that the first principal component (Dim1) explained 60.9% of the total variance and represented a general activation gradient of sporulation-related genes (Fig. 2A). All genes were positively correlated with Dim1, indicating that this axis reflects the overall transcriptional engagement in sporulation. Dim2, which accounted for only 13.6% of the variance, provided limited discrimination and was not further interpreted.

The PCA score plot revealed three main visual groups that corresponded to distinct phases of sporulation gene expression across pH conditions (Fig. 2B). The first group is composed of samples with minimal or no expression of sporulation-related genes. This group included all time points from early samples (*t*  ≤  20 h) from all three sporulation conditions, samples from pH 7.0 to 5.5 collected for 30–50 h, and all pH 7.0–4.0 time points, indicating no initiation of sporulation. The second group represented the onset of sporulation, as reflected by the initial activation of key genes. Samples from pH 7.0 entered this group as early as 20–30 h, whereas samples from pH 7.0–5.5 only joined this cluster from 50 h onward, suggesting a transcriptional delay of approximately 20–30 h following the acidification. The third group corresponded to maximal gene expression during active sporulation. It included samples from the pH 7.0 condition between 48 and 100 h and from pH 7.0–5.5 between 65 and 117 h, further reinforcing the temporal shift in transcriptional dynamics. Samples from pH 7.0 collected at 144 h appeared to return towards the first cluster, reflecting a downregulation of sporulation genes once spore formation was completed.

**Fig 3:**
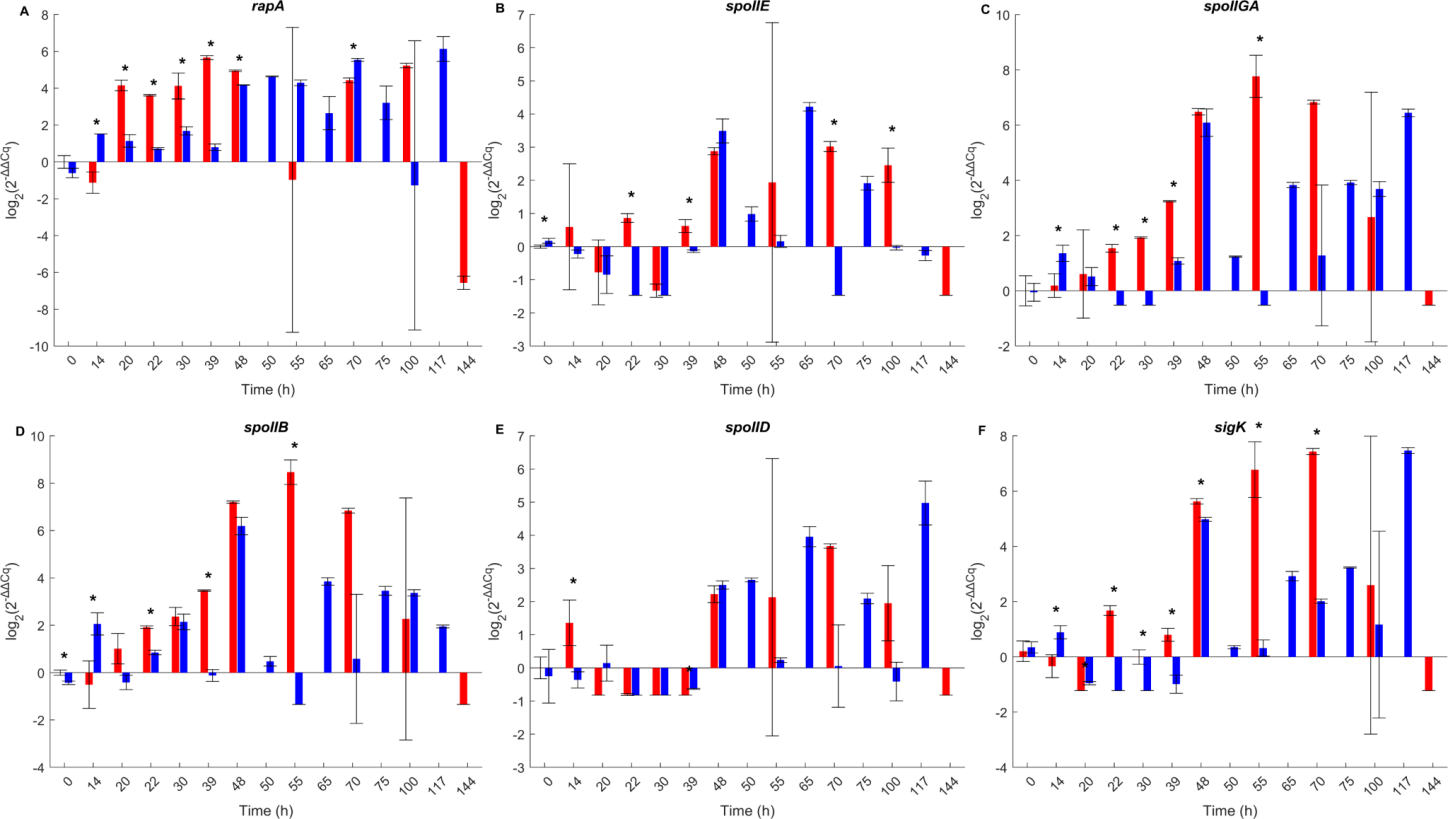
Temporal expression profiles of sporulation-related genes. Bar plots represent the log_2_(2^–^*^ΔΔCq^*) values of six key sporulation genes over time under pH 7.0 (red) and pH 7.0–5.5 (blue) conditions: *rapA* (**A**), *spoIIE* (**B**), *spoIIGA* (**C**), *spoIIB* (**D**), *spoIID* (**E**), and *sigK* (**F**). Each bar represents the mean of two biological replicates, each measured in technical duplicate, with error bars representing the standard deviation. Asterisks (*) indicate significant differences (*P* < 0.05) between the two conditions at each corresponding time point.

The temporal expression patterns of six key regulators were analyzed individually using the log_2_(2^–*ΔΔCq*^) values (Fig. 3). Under static pH 7.0, all genes exhibited a sequential and coordinated activation pattern consistent with the known stages of the sporulation cascade. Early-stage genes such as *rapA*, *spoIIE*, *spoIIGA*, and *spoIIB* showed significant upregulation from 20 to 22  h post-inoculation, with expression peaking between 48 and 70  h. The mid-stage gene *spoIID* reached its maximum expression between 55 and 65  h. The late-stage sigma factor *sigK* showed strong expression from 55 to 100 h before decreasing at 144  h. Under pH 7.0–5.5, all six genes followed the same sequence of activation but displayed delayed and prolonged expression profiles. A first transient peak of expression was observed at 48  h for *spoIIE*, *spoIIGA*, *spoIIB*, and *sigK*, followed by a marked decrease and a second wave of expression starting from 65  h. Expression of *spoIIGA* and *sigK* peaked at 117  h. For *rapA*, significant expression was detected from 48  h onward and remained sustained until 117  h. The expression of *spoIID* under this condition also increased gradually from 65  h and remained elevated through the late time points. Across all genes, the duration of expression was broader under pH 7.0–5.5, with activation generally starting later and lasting longer compared to pH 7.0. By contrast, under pH 7.0–4.0, none of the genes reached quantifiable expression levels after the shift, and expression remained below detection thresholds throughout the time course.

For the stress and sporulation regulation-related genes *disA*, *des*, and *rsiO*, expression remained predominantly downregulated over time across all pH conditions, with no marked increase compared to the initial time point (0 h) (results not shown).

### Spore properties

The evaluated spore properties included heat resistance and germination ability. These assessments were conducted on *B. subtilis* spores produced under different pH conditions. However, the pH 7.0–4.0 condition did not yield a sufficient quantity of spores for reliable analysis of heat resistance and germination ability (approximately 2.5 log_10_ spores.mL^−1^). Consequently, the experiments were performed only on spores obtained from the pH 7.0 and pH 7.0–5.5 conditions.

#### Heat resistance

**Fig 4:**
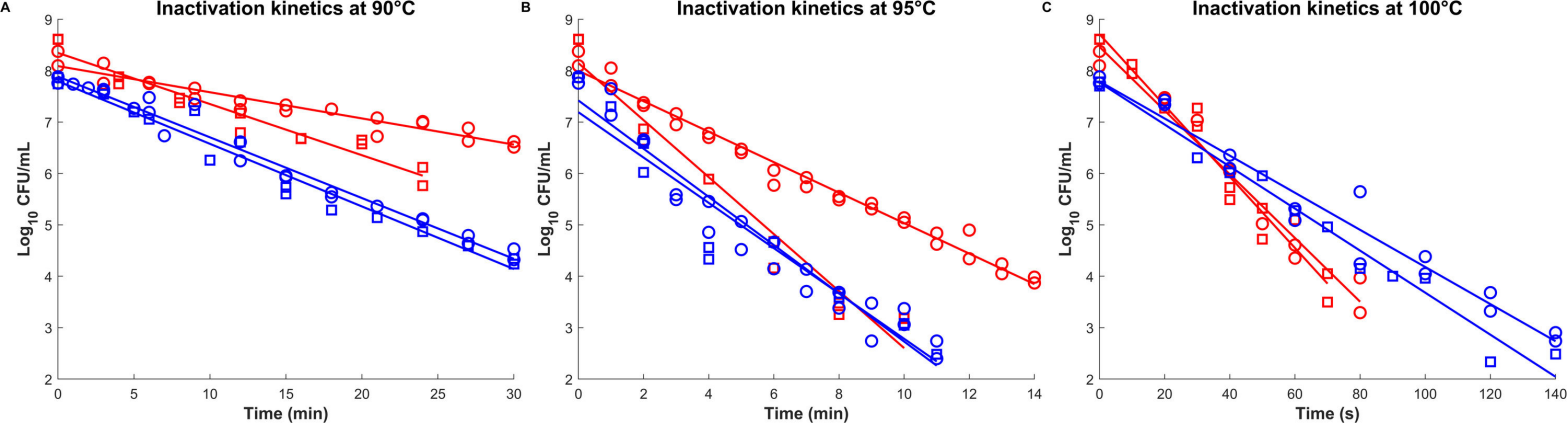
Inactivation kinetics at 90°C (**A**), 95°C (**B**), and 100°C (**C**) of spores of the pH 7.0 (red) and pH 7.0–5.5 (blue) conditions. The lines correspond to the fit of the modified Weibull model, based on all individual technical replicates from both biological replicates represented by circles and squares.

The inactivation kinetics of spores produced under static pH 7.0 and pH 7.0–5.5 conditions were studied at 90°C, 95°C, and 100°C (Fig. 4). The survival curves were fitted using a simplified Weibull model. The likelihood test confirmed that fixing the shape parameter *p* at 1 does not negatively impact the fit quality; therefore, the heat inactivation curves were modeled with a fixed *p* = 1. Low AICc and RMSE values indicate a good fit to experimental data. Statistical analysis indicated that log_10_(*D-*values) were generally comparable between the two pH conditions. In addition, no significant differences in *D-*values were observed between biological replicates (R1 vs R2), indicating good reproducibility of the heat resistance assays. The log_10_(*D-*values) at the reference temperature (100°C) was −0.31 for spores of the pH 7.0 condition and −0.46 for the spores of pH 7.0–5.5 conditions, with a global *Z*-value of 6.89°C.

#### Germination abilities

**Fig 5:**
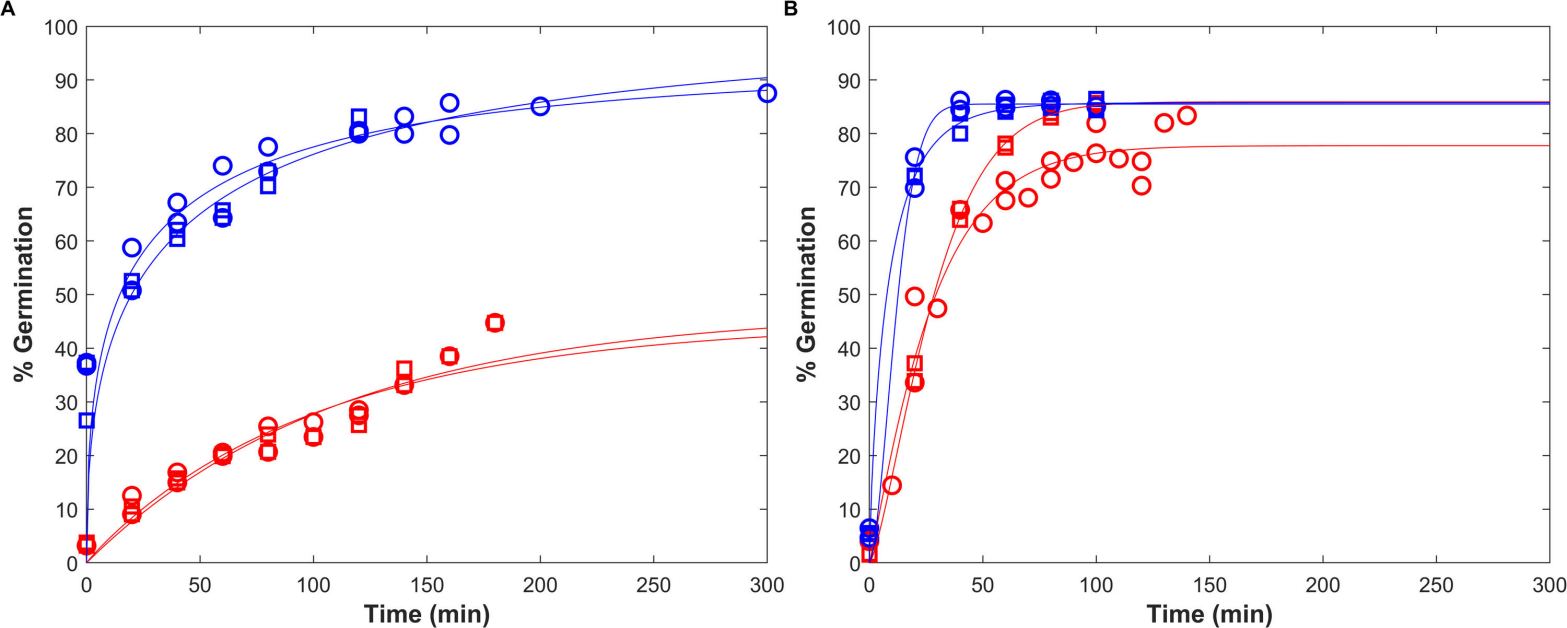
Germination percentage kinetics of spores produced at two different pH conditions: at pH 7.0 (red) and pH 7.0–5.5 (blue), and incubated in L-alanine suspension (**A**) and BHI medium (**B**). The solid lines correspond to the fit of the Weibull model, based on all individual technical replicates from both biological replicates represented by circles and squares.

**Fig 6:**
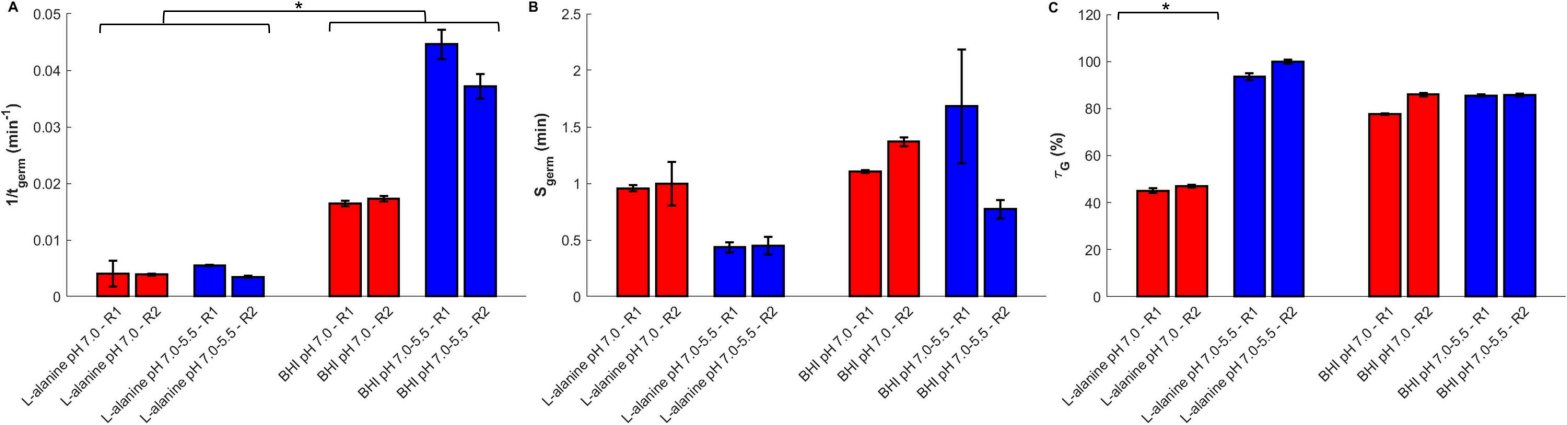
Germination parameters of spores produced at two different pH conditions: at pH 7.0 (red) and pH 7.0–5.5 (blue). (**A**) The inverse of *t*_germ_ (1/*t*_germ_) represents the germination rate, with higher values indicating faster germination. (**B**) *S*_germ_, the scattering parameter, reflects the variability in germination within the population. (**C**) *τ_G_* (% germination efficiency) represents the final proportion of spores that completed germination. Error bars represent the estimated standard deviation between the two biological and technical replicates; statistically significant differences (*P* < 0.05) are marked with an asterisk (*).

The germination kinetics of spores were evaluated in two different media (BHI and L-alanine suspension) and at two sporulation pH levels (pH 7.0 and pH 7.0–5.5) (Fig. 5). The germination curves were fitted using a modified Weibull model, and the three key germination parameters were extracted: *t*_germ_ (time taken to reach 90% of maximum germination), *S*_germ_ (scattering parameter), and *τ_G_* (final germination proportion). Low AICc and RMSE values indicated a good fit to experimental data. Significant effects of the germination medium and the sporulation pH condition on the germination abilities of spores were observed (Fig. 6). The germination rate (1/*t*_germ_) was significantly higher in BHI compared to L-alanine (*P* = 0.028), for the two sporulation pH conditions, suggesting that BHI promotes faster germination. However, no significant difference in germination time was observed between pH 7.0 and pH 7.0–5.5 within L-alanine (*P* = 1.000) or BHI (*P* = 0.333), indicating that sporulation pH conditions tested alone do not significantly impact germination time within the same germination medium. The scattering parameter (*S*_germ_) did not show significant differences among conditions (*P* > 0.05), suggesting that the heterogeneity of the germination response remains similar regardless of the germination medium or sporulation pH. By contrast, the final germination proportion (τ*_G_*) was significantly lower in L-alanine for the spores produced at pH 7.0 compared to the pH 7.0–5.5 condition (*P* < 0.05) (Fig. 6C). In addition, no significant differences were observed between biological replicates (R1 vs R2) for any condition (*P* = 1.000), indicating good reproducibility of the germination assays.

#### Effect of sporulation under pH shift conditions on the expression of genes related to spore properties

**Fig 7:**
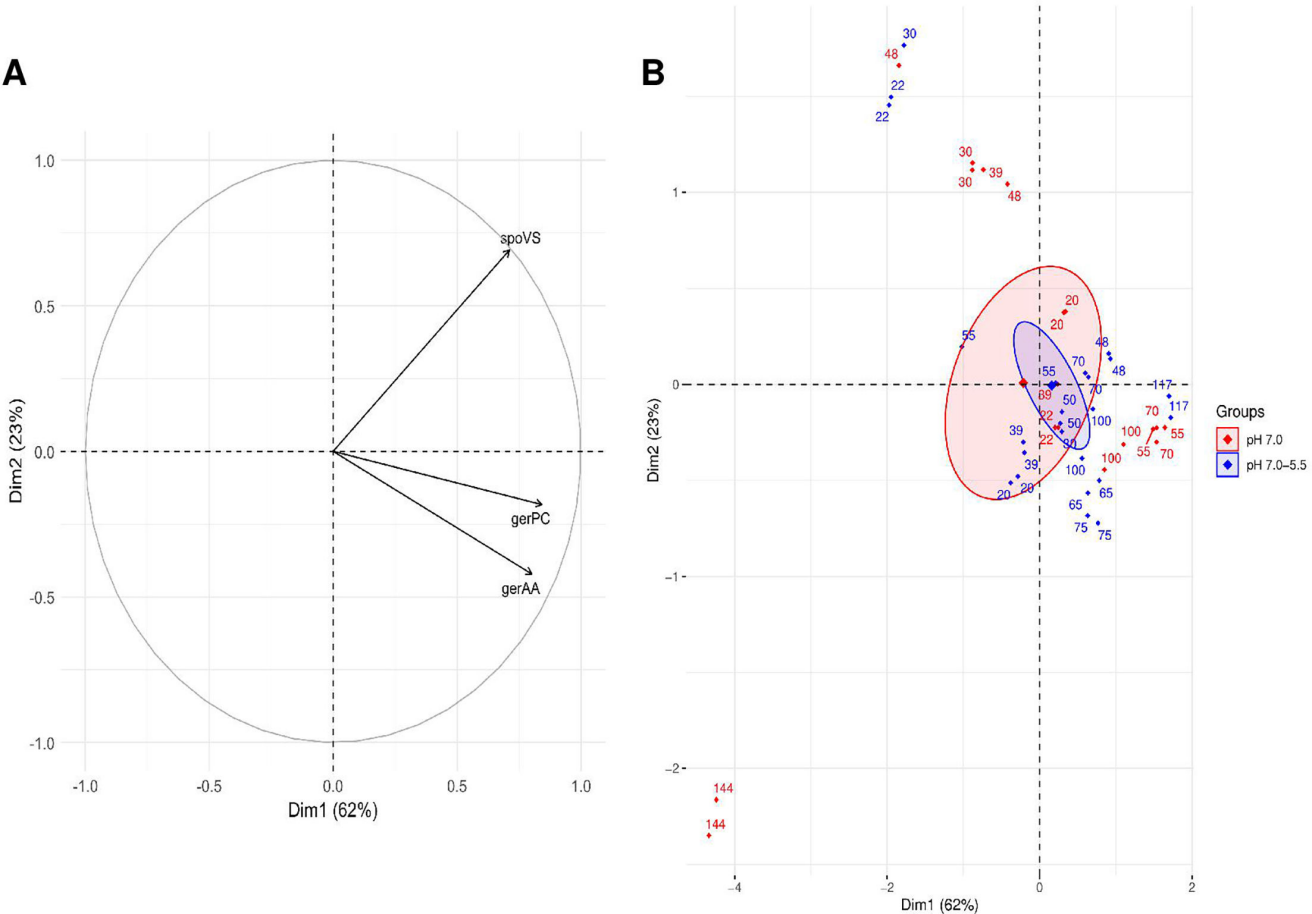
PCA of spore properties related genes expression produced under pH 7.0 and pH 7.0–5.5 conditions, performed using only time points after the pH shift (≥16 h). (**A**) Correlation circle of selected genes involved in spore germination and spore core dehydration, illustrating their contributions to the first two principal components (Dim1 and Dim2). (**B**) PCA score plot displaying sample distributions based on their gene expression profiles under pH 7.0 (red) and pH 7.0–5.5 (blue) conditions. Each point corresponds to one of the two biological replicates at a given time point and represents the mean of two technical replicates. Time points are labeled (≥16 h to 144 h, depending on the time at which the sampling was performed), and 95% confidence ellipses illustrate group dispersion by sporulation condition.

The correlation circle (Fig. 7A) shows that all three genes contribute distinctly to the principal components. Dim1 (62% of the variance) is mainly associated with *gerPC* and *gerAA*, both involved in spore germination, whereas Dim2 (23% of the variance) is primarily influenced by *spoVS*, which plays a role in spore coat assembly and core dehydration. The PCA score plot (Fig. 7B) reveals no significant differences between pH 7.0 (red) and pH 7.0–5.5 (blue) conditions.

Data from pH 7.0–4.0 were not included in the PCA. However, it is important to note that for this condition, the quantification of *spoVS*, *gerPC*, and *gerAA* after the pH shift was below the limit of detection, suggesting minimal or no expression under these conditions.

## DISCUSSION

The results of this study confirm that sporulation efficiency is highest at pH 7.0 and declines as pH shifts away from this optimal value, consistent with previous findings conducted under static pH conditions (13, 14, 17, 20). Importantly, this study also demonstrates that pH shifts significantly impact sporulation kinetics. The effect of these shifts depends on whether the pH remains within the growth-permissive range or drops below the minimum pH required for growth. In the pH 7.0–5.5 condition, sporulation was delayed but ultimately reached similar spore concentrations as those observed at pH 7.0. This indicates that the transition to a lower pH slowed the sporulation process without fully inhibiting it. Although studies on the impact of pH shifts on sporulation are lacking, one study on *Clostridioides difficile* strain 630 reported that a 1.0-unit decrease in pH, dropping from approximately pH 6.8 to 5.8 during cultivation, delayed growth by approximately 3 h (55). While *C. difficile* strain 630 can grow within a broader pH range of approximately 5.5 to 9.0, this finding highlights how even moderate acidification within the growth-permissive range can significantly affect cellular processes (55). Given that growth and sporulation exhibit similar pH sensitivities (13, 14, 16, 17, 20), our results suggest that a comparable mechanism may also apply to sporulation in *B. subtilis*. In this case, the shift to pH 5.5 may have temporarily suppressed sporulation activity, leading to a delay before the process resumed without being entirely blocked. By contrast, the pH 7.0–4.0 transition led to complete inhibition of sporulation, indicating that exposure to an environment beyond the growth limits of *B. subtilis* (17) resulted in severe stress, preventing differentiation into spores. Instead, vegetative cells likely underwent inactivation, as shown in the growth kinetics. This observation is consistent with findings from the same study on *Clostridioides difficile*, where a 1.5-unit pH decrease, dropping from approximately pH 6.8 to 5.3 (pH outside the growth limit of *Clostridioides difficile*), fully inhibited growth (55). The model accurately reflected this observation by estimating a delayed *t*_max_ at pH 5.5 compared to pH 7.0, inducing the observed delay in sporulation kinetics, and by setting sporulation parameters to zero at pH 4.0, confirming that sporulation could not occur under these conditions. However, one limitation of the current model is that it does not account for vegetative cell inactivation, and thus cannot simulate the observed decline in viable cells following the shift to pH 4.0. Integrating a viability loss function would enhance future model predictions under extreme stress conditions.

The gene expression analysis provided complementary insights into the phenotypic sporulation kinetics and reinforced key assumptions embedded in the sporulation model. By combining PCA and gene-by-gene time-course profiling, we were able to characterize how the sporulation program was modulated under pH shift conditions and to clarify the transcriptional basis for the observed delays in spore formation.

Before the pH shift (*t* ≤ 14  h), gene expression profiles were similar across all conditions. This was reflected in the PCA, where early samples from pH 7.0, pH 7.0–5.5, and pH 7.0–4.0 were tightly clustered, indicating no transcriptional divergence during early growth. After the acidification, transcriptional divergence emerged, confirming that the pH shift was the driver of subsequent changes in sporulation dynamics.

Under pH 7.0, samples followed a smooth and continuous trajectory in PCA space along Dim1, reflecting progressive and well-coordinated activation of sporulation genes. By contrast, samples from pH 7.0 to 5.5 shared a similar trajectory but were shifted temporally. Initial gene activation was delayed, and samples entered the zone of strong gene expression only from 50  h, whereas pH 7.0 samples reached this stage as early as 20–30  h. This suggests a transcriptional delay of approximately 20–30  h, consistent with the observed phenotypic delay in spore accumulation and with the model-estimated time to achieve the maximum sporulation probability (*t*_max_).

Under pH 7.0–4.0, all analyzed sporulation genes remained below detection levels after the shift, confirming transcriptional arrest and supporting the model assumption of the maximum sporulation probability (*P*_max_) of 0 under such acidic stress.

The gene expression dynamics also confirmed the canonical order of sporulation events. At pH 7.0, early-stage genes (*spoIIE*, *spoIIGA*, *spoIIB*) were activated at 22 h, followed by the mid-stage engulfment gene *spoIID* at 48 h and the late-stage sigma factor *sigK* between 48 h and 100 h. These timings are consistent with previous morphological studies (36, 56) and demonstrate that, under optimal conditions, transcriptional and morphogenetic programs are tightly coordinated. Under pH 7.0–5.5, the same sequence of gene activation was preserved, although delayed, suggesting that the sporulation process remained functional despite the acid stress. Several genes displayed a biphasic expression pattern, with an initial peak, a transient decline, and a subsequent reactivation at later time points. This behavior may indicate a temporary interruption followed by a re-initiation of the sporulation program after cellular adaptation to the new pH conditions. One potential contributing factor is the sustained expression of *rapA* observed after the pH shift. RapA is a phosphatase that dephosphorylates Spo0F ~ P, thereby reducing the phosphorylation of Spo0A, the master regulator required to initiate sporulation gene expression (36). Although RapA does not directly repress transcription, its regulatory role on the phosphorelay cascade can indirectly delay or attenuate the transcription of key sporulation genes (*spoIIE*, *spoIIGA*, *sigK*, etc.) by maintaining Spo0A in its inactive form (36, 57, 58). Further investigations using Spo0A ~ *P* quantification or single-cell approaches could help confirm whether Spo0A activation dynamics align with these transcriptional patterns.

Finally, in this study, we assumed a fixed spore formation time (*t_f_*) of 7 h in the model, based on established literature (46). While the transcriptional data offered good resolution on the onset of gene activation, they could not provide accurate estimates of the time needed to complete sporulation after commitment. Our attempt to infer *t_f_* using RT-qPCR data was inconclusive, highlighting the limitations of population-level averages for such dynamic and heterogeneous processes. Future studies using single-cell technologies will be needed to precisely resolve the timing of sporulation commitment and completion at the individual cell level (59, 60).

Beyond *B. subtilis*, the modeling approach developed here has the potential to be adapted to other food-relevant spore-forming bacteria. The model under static conditions has already been successfully applied to *Bacillus licheniformis* (61). A critical step in adapting the model to other species is the accurate estimation of the time of spore formation (*t_f_*), which is likely to vary depending on physiological and ecological traits, like the other sporulation parameters.

In addition, although this study implemented a single-step pH shift, this controlled scenario lays the groundwork for modeling more complex environmental changes. In real food processing systems such as fermentation, pH often evolves gradually or in multiple phases over time. The modeling framework developed here can accommodate multiple successive pH shifts, enabling future simulations of sporulation under more realistic and fluctuating conditions.

Beyond its effect on sporulation kinetics, pH significantly influences spore properties, including heat resistance and germination ability (20, 23, 33, 55, 62). In our study, we observed that spores formed under the static pH 7.0 and pH 7.0–5.5 conditions exhibited comparable heat resistance, indicating that the shift to pH 5.5 did not affect the heat resistance of the produced spores. In addition, germination assays revealed that these spores germinate significantly faster in Brain Heart Infusion (BHI) medium, regardless of the sporulation pH condition. The enhanced germination rate observed in BHI medium can be attributed to its rich composition of various nutrients that act as germinants, facilitating non-specific germination pathways. BHI contains a complex mixture of amino acids, sugars, and other growth factors (63) that can collectively trigger spore germination through multiple germinant receptors (61). This non-specific activation contrasts with germination in L-alanine suspension, which primarily targets receptors encoded by the *gerA* operon (64, 65). Interestingly, in L-alanine suspension, spores formed under the pH 7.0–5.5 condition reached a higher final germination percentage than those produced at static pH 7.0, highlighting a strong improvement in their germination potential. This suggests that pH variations during sporulation can enhance the responsiveness of spores to specific germinants, possibly by modulating the expression or functionality of key germinant receptors such as GerA (64, 65). These findings emphasize the combined influence of the germination medium and the sporulation pH on the germination capacity of *Bacillus subtilis* spores.

The effect of sporulation pH on spore heat resistance has been widely studied; however, findings in the literature remain contradictory. Some studies indicate that sporulation at acidic pH decreases heat resistance while maintaining *Z*-values (15, 19, 21). For instance, *Bacillus cereus* spores show a 65% reduction in *D*-values per pH unit increase, indicating that sporulation under acidic conditions compromises heat resistance (15). Similarly, *B. weihenstephanensis* KBAB4 spores formed at pH 5.9 exhibited nearly 50% lower *D*_90°C_ values compared to those formed at pH 7.2 (19). On the other hand, some studies report that spores formed at acidic pH can exhibit higher wet-heat resistance (14, 20), suggesting that the relationship between sporulation pH and heat resistance is complex and may depend on the strain, the methodology used, and the pH tested. One of the main challenges in comparing our results to previous studies is that most of them examined spores formed under static conditions, while this study investigated sporulation under pH shift conditions. To our knowledge, no studies have specifically addressed the impact of pH shift changes on spore properties. While the sporulation kinetics suggest that most spores in the pH 7.0–5.5 condition were formed at pH 5.5, a key question arises concerning the impact of the pH at which vegetative growth occurred on the subsequent heat resistance of spores. A study by Baril et al. demonstrated that the wet-heat resistance of *B. weihenstephanensis* KBAB4 spores produced in a two-step sporulation process depended on the sporulation temperature but not on previous cell history (19). However, this conclusion was drawn for temperature effects, and data on the influence of initial pH conditions on subsequent spore resistance are lacking. Furthermore, many studies focus on the initial pH of the sporulation medium, even though pH fluctuations naturally occur during sporulation, even in buffered conditions. For example, Mazas et al*.* reported that sporulation pH can shift from 6.0 to 8.2 or from 5.9 to 8.0, depending on *B. cereus* strains, potentially influencing final spore resistance (15).

A similar challenge applies to the effect of pH on germination efficiency. Previous studies have shown that sporulation pH alters spore biochemical composition and consequently affects germination potential. For instance, *Clostridium perfringens* spores produced at pH 8 exhibit the highest germination efficiency, whereas further pH increases reduce germination while enhancing resistance to wet heat (18). Likewise, *Thermoanaerobacterium thermosaccharolyticum* spores demonstrate the highest post-heat treatment recovery rates at pH values near optimal growth (6.0–6.5), while extreme pH values lead to lower recovery rates (33). However, as with heat resistance, these studies mainly focus on static pH conditions, making direct comparisons with shift scenarios challenging.

In this study, we analyzed the expression of genes associated with spore properties to better understand the molecular mechanisms underlying the observed phenotypic differences in heat resistance and germination efficiency. PCA of gene expression revealed no clear separation in the expression profiles of *spoVS* (involved in spore coat assembly and core dehydration), *gerPC*, and *gerAA* (encoding a subunit of the germination receptor complex GerA, responsible for sensing nutrient germinants such as L-alanine) (37, 46) between the pH 7.0 and pH 7.0–5.5 conditions. This indicates that, at the transcriptional level, the expression of these genes was not significantly affected by the acidification from pH 7.0 to pH 5.5 during the tested time points.

However, despite the absence of significant transcriptional differences, spores produced under pH 7.0–5.5 displayed enhanced germination efficiency in the L-alanine suspension compared to those formed under pH 7.0. This significant difference suggests that regulatory mechanisms beyond gene transcription may modulate germination potential. A possible explanation involves post-transcriptional regulation, which could influence the actual protein levels of GerA components without affecting transcript abundance (36).

Taken together, this study provides insights into how pH shifts affect sporulation kinetics, spore properties, and gene expression. However, all analyses were conducted at the population level, which can hide important cell-to-cell heterogeneity. Such variability is well documented in sporulation initiation, spore resistance, and germination behavior and may be amplified under stress (66–68). To capture this diversity, future studies should implement single-cell or spore-resolved techniques, such as time-lapse microscopy, flow cytometry, or single-cell transcriptomics (69, 70), to refine models and improve the mechanistic understanding of sporulation under fluctuating conditions.

### Conclusion

This study provides new insights into how *Bacillus subtilis* adapts its sporulation dynamics and spore properties in response to pH shift conditions. Through the integration of experimental data and a predictive growth-sporulation model, we demonstrated that a pH shift from 7.0 to 5.5 leads to a significant delay in sporulation, while a shift to pH 4.0 completely blocks the process. These findings confirm that pH transitions, even within the growth-permissive range, can disrupt the timing of sporulation, delaying the transcriptional commitment of the population without compromising the sporulation cascade’s integrity. The model effectively captured these dynamics by adjusting the *t*_max_ and setting *P*_max_ to zero under non-permissive conditions.

Gene expression analysis reinforced these observations. Under the pH 7.0–5.5 condition, although the overall trajectory of sporulation gene activation was preserved, expression was delayed by 20–30 h, depending on the gene, in alignment with the phenotypic lag in spore formation. A transient, unsustained expression peak shortly after the shift suggests that a subpopulation may have initially attempted to commit to sporulation before being suppressed by environmental stress. Importantly, despite transcriptional delay, the canonical sequence of gene activation was maintained, highlighting the robustness of the sporulation network under moderate acid stress. Conversely, gene expression at pH 4.0 was strongly repressed post-shift, validating the assumption that sporulation cannot occur under severe acidification.

Regarding spore properties, we found that spores produced under the pH 7.0–5.5 condition exhibited enhanced germination in L-alanine despite no major differences in the transcription of germination-related genes such as *gerAA* and *gerPC*. This uncoupling between transcript levels and phenotype suggests that post-transcriptional or post-translational mechanisms may modulate spore responsiveness to germinants. These hypotheses call for further investigation using proteomics or single-cell transcriptomic tools.

Overall, this study offers a comprehensive perspective on how acid stress affects the entire *B. subtilis* life cycle, from vegetative growth to sporulation, spore germination, and heat inactivation. The predictive model developed provides a foundational framework for simulating more realistic scenarios encountered in food processing or environmental systems, where pH fluctuations are common. Understanding how sporulation kinetics and spore traits respond to such changes is essential for designing improved microbial control strategies. In future work, incorporating other environmental factors (e.g., temperature shifts, water activity) and applying single-cell technologies will help refine our models and further unravel the complex regulation of sporulation.

## Data Availability

All data supporting the findings of this study, including sporulation kinetics, gene expression measurements, heat resistance, and germination abilities, have been deposited in the Zenodo repository and are publicly available under the DOI: https://doi.org/10.5281/zenodo.16736469.
